# Cuproptosis and Cardiovascular Diseases: Mechanisms, Pathophysiology, and Therapeutic Strategies—A Narrative Review

**DOI:** 10.31083/RCM38833

**Published:** 2025-09-18

**Authors:** Zhongkai Wang, Changyong Wu, Ruijie Li, Huang Sun, Menghan Li, Yihua Luo, Suli Bao, Yunzhu Peng

**Affiliations:** ^1^Department of Cardiology, The First Affiliated Hospital of Kunming Medical University, 650032 Kunming, Yunnan, China; ^2^Cardiovascular Disease Center, The Affiliated Hospital of Yunnan University, 650021 Kunming, Yunnan, China

**Keywords:** cuproptosis, cardiovascular diseases, molecular mechanism, oxidative stress, therapeutic strategies

## Abstract

Despite recent efforts and improvements in terms of diagnosis and treatment, cardiovascular diseases (CVDs) remain a prime risk factor for mortality globally; thus, elucidating novel mechanisms underlying the development of these diseases remains essential. There have been significant contributions to identifying the classical means of programmed cell death (PCD), such as apoptosis, necroptosis, pyroptosis, and autophagy, in CVDs. In comparison, although the role of cuproptosis in CVDs is relatively unknown, cuproptosis has recently been revealed as a distinct type of copper-induced cell death with a unique molecular signature and regulation compared to conventional forms of PCD. Thus, cuproptosis represents a novel approach for treating CVDs. To investigate such implications in this review, we will systematically study the cellular mechanisms of cuproptosis and its pathophysiological roles in various forms of CVD. Finally, based on such mechanistic knowledge and to bridge mechanistic research with clinical applications, we propose the use of therapeutic strategies such as copper chelation, antioxidant modalities, and ferredoxin 1 (FDX1)/lipoic acid synthetase (LIAS)-based biomarkers.

## 1. Introduction

With cardiovascular diseases (CVDs) accounting for an estimated 20 million deaths a year and a further rising burden on health care [1, 2], they are the leading cause of death worldwide. The burden of CVDs has increased by an astounding 60% over the last 30 years. This growth will occur despite the enormous “healthcare burden” created by CVDs across the full national developmental continuum with CVDs also generating what is currently the rising mortality rates and healthcare demand on health system resources. An increase in both these factors simultaneously occurs and remains to be resolved, decoding the more multifactorial pathophysiological contributions. An increasing number of reports have confirmed that trace element dysregulation, such as deficiency or toxic overload, plays a vital role in CVDs [3, 4, 5] and strongly influences disease pathogenesis. In this context, copper plays a pivotal role in regulating key physiological processes spanning from mitochondrial bioenergetics through electron transport chain (ETC) modulation to homeostatic ionic balance and the transduction of metabolic energy [6].

Copper exerts bidirectional regulatory effects on cardiovascular homeostasis through various pathological cascades. Deficient copper bioavailability disrupts cardiogenesis and lipidomic networks while inducing anaemic syndromes that exacerbate myocardial dysfunction via compromised oxygen transport and altered haemodynamic equilibrium [7]. Conversely, copper overload induces reactive oxygen species (ROS) generation via the Fenton reaction, which activates the nucleotide-binding domain leucine-rich repeat containing protein 3 (NLRP3) inflammasome and triggers the release of pro-inflammatory cytokines such as interleukin-1β (IL-1β), culminating in a self-perpetuating “oxidative stress‒inflammation” vicious cycle, synergistically fostering the progression of CVDs [8, 9].

Prior work on a copper-dependent programmed cell death (PCD) process suggests potential implications for cardiovascular ageing due to shared mitochondrial dysfunction mechanisms in cancer models, although direct evidence in ageing hearts remains scarce. This degenerative pathophysiology can be described as a tripartite metabolic disease that results from a breakdown of cellular iron chelation mechanisms, irreversible mitochondrial structure remodelling and protein lipoylation-dependent phase transformation of proteins. These impairments lead to the breakdown of toxic iron-sulfur (Fe-S) clusters, resulting in impaired oxidative phosphorylation and metabolic plasticity in neuronal electron transfer complexes [10, 11].

Growing evidence has confirmed that cuproptosis could be a therapeutically targetable form of cell death in oncological, neurological and metabolic diseases [7, 12, 13], the modifiability of which has been confirmed in several experimental models. Nevertheless, cuproptosis represents a significant perspective in the field of oncology, whereas the exact pathways involved in its regulation in the context of the cardiovascular system are yet to be elucidated. Thus, in this review, the mechanistic participation of cuproptosis in the pathophysiology of CVDs has been systematically reviewed along with molecular mechanisms, including ferredoxin 1 (FDX1)-mediated mitochondrial cuproptosis, therapeutic and translational potential, and bidirectional tactics in copper overload, with the aim of extending the application of personalized cardiovascular medicine.

## 2. Mechanisms of Cuproptosis

We present next a step-by-step approach to understanding the mechanism whereby cuproptosis contributes to CVDs: the contribution of cuproptosis involves multiple layers of biological mechanisms. We discuss first the history of discovery of cuproptosis from the observation of copper cytotoxicity to its description as a specific type of cell death. Accumulated knowledge on its biological aspects is essential to building a conceptual view explaining the functional differences of cuproptosis from canonical PCD modalities. Here, we use comparison of their biochemical identities to describe how the biochemical traits of cuproptosis differ from the classical PCD pathways regarding distinct copper-dependent activators and effectors. We then move on to describe systemic copper homeostasis by describing the processes of intestinal absorption, chaperone-mediated trafficking, and hepatobiliary excretion that form the networks by which the bioavailability of a metal is determined. Ultimately, the cascade that leads to a failure of the homeostatic regulatory network and thus eventually copper overload, which is pivotal in the pathological mechanisms in ferroportin knockout animals. Specifically, overload with copper results in aggregation of lipoylated enzymes and loosening of Fe-S clusters to cause mitochondrial proteotoxicity.

### 2.1 The Discovery of Cuproptosis

Chan *et al*. [14] first showed in 1978 that high concentrations of intracellular Cu^2+^ can cause cytotoxicity in fibroblasts, which initiated systematic studies on the involvement of copper in PCD. The early-era research used genetic models of copper transport defects such as Menkes disease and Wilson’s disease to characterize three features of copper toxicity as intracellular overload, metalloprotein trapping, and impairment of the mitochondrial respiratory process [15, 16, 17, 18]. In this regard, work later identified saved mechanistic conservation between the cell death by copper-induced toxicity with the pathologic mechanistic causes of neurodegeneration (protein misfolding cascade) and of cancer (metabolic reprogramming) [19, 20], which took conceptual unification in 2022 with the molecularly defining by Tsvetkov and colleagues [10] of this pathway, which was termed “cuproptosis” and supported by multiomics.

### 2.2 Copper Metabolism

Copper is an essential trace element in humans, and its regulation of copper homeostasis is ensured in human physiology by dedicated transporters and chaperones. This trace element is an important catalytic cofactor of biosynthetic processes that synthesize vital biomolecules, such as neurotransmitters (dopamine and epinephrine), mitochondrial ETC (cytochrome C oxidase, CCO), antioxidant defence (superoxide dismutase 1, SOD1) and oxygen transport complexes (haemoglobin), to maintain neurosignalling, bioenergetics and respiration [7]. Copper metabolism involves a homeostatic balance of multiple steps of the regulation of ionic pools and the enzymes responsible for permitting nutritional intake, copper intracellular distribution, biochemical activation and, ultimately, systemic clearance [21]. Nutritional intake, which leads to their absorption, is the main step for small intestine enterocytes and the duodenal and proximal jejunum, and it is supported by enterohepatic recycling mechanisms with maintenance of the systemic pool [22, 23]. Intestinal absorption involves copper transporter 1/solute carrier family 31 member 1 (CTR1/*SLC31A1*) and copper transporter 2/solute carrier family 31 member 2 (CTR2/*SLC31A2*), which can mediate the influx of Cu^+^ in the apical membrane, whereas divalent metal transporter 1/solute carrier family 11 member 2 (DMT1/*SLC11A2*) mediates limited Cu^2+^ influx in yet unidentified trafficking processes, where most of the Cu^2+^ is reduced to Cu^+^ by metalloreductases such as the six-transmembrane epithelial antigen of the prostate (STEAP) on the surfaces of gastric and duodenal epithelial cells [24, 25]. Copper absorption efficiency varies depending on whether the rate of dietary copper bioavailability can be modified by competing cations (iron/zinc) as well as by cellular redox situations and the availability of transporter profiles [26].

Life forms maintain accurate copper distribution via specific copper-containing molecular chaperones and delivery systems. Cytosolic copper chaperones, such as antioxidant 1 copper chaperone (ATOX1), CCO copper chaperone 17 (COX17) and copper chaperone for superoxide dismutase (CCS), orchestrate targeted delivery of copper ions to their target compartments and prevent cellular toxicity [27]. ATOX1 is a master copper transporter involved in intracellular copper trafficking that interacts directly with the P-type adenosine triphosphatases (ATPases) including ATPase copper-transporting alpha (ATP7A) and ATPase copper transporter beta (ATP7B) [28]. These membrane transporters actively sort and redistribute among the trans-Golgi network, plasma membrane and cytoplasmic vesicles to regulate copper flux. Tissue-specific expression patterns show that ATP7A dominates the hepatic and intestinal systems, whereas ATP7B displays predominantly hepatic and cerebral expression [29]. The copper-transferring activity of ATOX1, facilitated by its complexation with ATP7A/ATP7B in the trans-Golgi network, provides the catalytic copper cofactor of core metabolic activities and redox homeostasis cuproenzymes (CCO and tyrosinase). Recent findings on the nuclear localization of ATOX1, together with copper-dependent binding to cell cycle regulators, suggest that ATOX1 may modulate cell cycle programs via the transcriptional machinery [30]. The equilibrium of copper in mitochondria hinges on COX17, which carries out an intermembrane metal transporter role facilitating the transport of metals to respiratory complexes; this chaperone works with the production of cytochrome C oxidase 1 (SCO1) and cytochrome C oxidase 2 (SCO2) assembly proteins to form functional CuA and CuB sites in CCO, permitting both enzymatic function and regulated mitochondrial copper storage [31, 32]. The antioxidant defence system is known, where CCS delivers copper to SOD1 for use in turning superoxides into less aggressive species and averting the associated oxidative pressure [33, 34]. The transport systems for controlling the systemic copper concentration include intestinal export by ATP7A, circulation delivery by ceruloplasmin, and biliary excretion by ATP7B, which together maintain a steady-state copper concentration throughout the body. They maintain vital copper-based physiology and regulate excessive metal deposition [29, 35].

### 2.3 Cuproptosis is Different From Known Modes of Cell Death

Cuproptosis is a newly discovered form of PCD that exhibits distinct molecular features compared with conventional types of cell death [36, 37, 38]. Although cuproptosis shares certain characteristics with apoptosis, such as mitochondrial dysfunction and cytochrome C release, its underlying mechanism differs significantly. Cuproptosis is initiated by copper ions binding to tricarboxylic acid (TCA) cycle proteins, leading to proteotoxic stress and Fe-S clusters depletion, rather than following the classical apoptotic pathway [10, 39, 40]. Unlike the caspase-mediated apoptotic cascades, cuproptosis triggers cell death by inducing metabolic protein complexes of lipids and metals, independently from classical apoptosis machinery [41]. Moreover, studies have revealed that cuproptosis and autophagy operate through distinct mechanisms. In autophagy, proteostasis is maintained via the recycling of damaged organelles, whereas cuproptosis triggers irreversible proteome destruction through copper-induced oligomerization of metabolic enzymes [42, 43, 44].

This work identifies cuproptosis as a copper-excess-mediated pathophysiological program characterized by proteotoxic stress and bioenergetic failure and illustrates the dual metabolic functions of copper, which is both an essential cofactor and a cytotoxic agent. This is an important mechanistic dualism that defines new points of therapeutic intervention for diseases characterized by copper dysregulation [45]. In addition to copper, other metals have been found to induce alternative pathways of PCD via different mechanisms; for example, ferroptosis is an iron-dependent pathway of cell death that is mediated by lipid peroxidation cascades, in combination with glutathione peroxidase 4 (GPX4) activity, glutaminolysis dynamics and redox-sensitive transcription factors, nuclear factor erythroid 2-related factor 2 (*NFE2L2*), tumour protein 53 (p53) and breast cancer 1-associated protein 1 (BAP1) [46, 47, 48, 49]. These putative factors act on each other and impact antioxidant responses, lipid metabolism and consequently the levels of cell vulnerability to ferroptosis. These related levels are evidenced by the fact that ferritin accretions are correlated with different diseases, such as cancer, degenerative diseases, CVDs and fibrosis [50, 51, 52, 53, 54], suggesting that this is a general principle of metallotoxicity. Furthermore, many ultrastructural characteristics of cuproptosis and ferroptosis overlap, such as mitochondrial swelling, plasma membrane integrity rupture, and cytoplasmic vacuolization [55], indicating that analogous cellular damage differs in two differently activated heavy elements and specific molecular triggers. The activation and development of CVDs are promoted by different metal elements that impact the imbalanced metabolic system in different manners.

### 2.4 Cuproptosis: FDX1-driven Lipoylation/Fe-S Cluster Failure Triggers Mitochondrial Collapse

Cuproptosis arises from Cu^+^ binding to lipoylated TCA cycle enzymes, triggering protein oligomerization, Fe-S cluster destabilization, and proteotoxic stress that culminate in cell death [10, 56]. This copper-dependent death pathway operates through three interdependent mechanisms: mitochondrial TCA cycle inhibition via enzyme inactivation and respiratory chain structural disassembly, leading to metabolic arrest and ETC failure; systemic metabolic collapse, characterized by impaired ATP synthesis; reduced nicotinamide adenine dinucleotide/flavin adenine dinucleotide (NADH/FADH_2_) generation; ROS overproduction; α-ketoglutarate depletion; and biosynthetic pathway disruption, causing amino acid/nucleotide deficits, redox imbalance, and toxic metabolite build-up under energy crisis conditions [57]. These three submechanisms converge to induce metabolic paralysis: mitochondrial dysfunction directly impairs energy production, systemic collapse amplifies oxidative damage, and biosynthetic failure prevents cellular repair. Critically, Fe-S cluster depletion serves as both a consequence and amplifier of this paralysis, exacerbating mitochondrial failure through dual pathways. Fe-S cluster depletion aggravates mitochondrial dysfunction through structural and chemical pathways. Structurally, the absence of the N2 cluster in complex I abolishes electron transfer from NADH to ubiquinone, terminating NADH oxidation and halting electron transport. Additionally, the Fe-S clusters in Complex I drive proton-pumping conformational changes, and their loss disrupts proton gradient formation and ATP synthesis [58]. Chemically, Cu^+^ amplifies Fe-S cluster degradation through Fenton reaction-derived hydroxyl radicals that oxidize sulfur ligands while competitively binding to assemble proteins such as Fe-S cluster assembly scaffold protein (ISCU) to block cluster biogenesis [59]. A tightly regulated enzymatic network comprising FDX1, lipoyltransferase 1 (LIPT1), lipoic acid synthetase (LIAS), dihydrolipoamide dehydrogenase (DLD), and dihydrolipoamide S-acetyltransferase (DLAT) coordinates lipoylation and TCA cycle functionality [60]. LIPT1 triggers specific redox inactivation of lipoyl (SRIL) dehydrogenase E2 subunits. LIAS functions to biosynthesize lipoic acid with S-adenosyl methionine (SAM)-catalysed sulfur transfer, and DLD recycles lipoamide, which is oxidized in multienzyme complexes, while DLAT promotes acetyl-CoA synthesis during pyruvate carboxylation. Notably, the loss of Fe-S clusters in Cu^+^-induced cells can occur via two inorganic pathways involving both a hydroxyl radical-assisted sulfur ligand oxidation process and active competition among cluster assembly proteins. Under physiological conditions, LIPT1 modifies target proteins with lipoic acid, LIAS produces lipoic acid, DLD maintains redox cycling in mitochondrial complexes, and DLAT supports acetyl-CoA synthesis [61, 62]. FDX1 is a mitochondrial [2Fe-2S] ferredoxin that donates electrons for steroid synthesis, lipoic acid production, and haem a biosynthesis. While it does not directly reduce copper, its iron-sulfur cluster is disrupted by the anticancer drug elesclomol-copper complex (Ele:Cu), linking FDX1 to copper-mediated toxicity in cells (Table 1, Ref. [8, 9, 10, 21, 30, 31, 59, 63, 64, 65, 66, 67, 68, 69, 70, 71, 72, 73, 74, 75, 76, 77, 78, 79]) [80]. The generation of Cu^+^ ions disrupts the physiological coupling of FDX1 and LIAS. In particular, the redox-active cysteine residue of FDX1 inhibits the Fe-S binding site of LIAS. Cys85 and Cys88 in FDX1 function together to drive electrons to LIAS to produce lipoic acid, an essential compound that activates the enzymes of the TCA cycle. In the presence of Cu^+^ ions, their reciprocity principle is inhibited, so the system is pushed further along the self-generating mechanism of metabolic breakdown, allowing sulfur assimilation into the lipoic acid precursor through a SAM-dependent mode to activate α-ketoacid dehydrogenase complexes, such as the pyruvate dehydrogenase complex (PDH), α-ketoglutarate dehydrogenase complex (KGDH) and branched-chain α-keto acid dehydrogenase complex (BCKDH), which regulate the consumption of TCA cycle substrates [63, 81, 82]. It may seem counterintuitive that the Cu^+^ ions produced by FDX1 activity inhibit the functional interaction with LIAS, perturbing the efficiency of lipoylation, particularly in the PDH complex, where deficient lipoylation destabilizes the thioester intermediate in the lipoyl domain. The resulting blockade of acetyl group transfer between the E1 and E2 subunits effectively halts decarboxylation activity, establishing this metabolic node as a critical fulcrum in cuproptosis execution [10, 83].

**Table 1.** **Experimental validation and category of cuproptosis labled core molecules in cardiovascular tissues**.

Category	Labeled molecules	Experimental phenotype	Refs.
Copper Transporters	CTR1	C57BL/6J mice	[69, 73]
	DMT1	No	[21, 79]
	ATP7A/ATP7B	None of the ATP7A; human of the ATP7B	[75, 76]
Copper Chaperones	ATOX1	No	[30, 71]
	COX17	No	[31, 65]
	CCS	No	[77]
Core Execution Proteins	FDX1	C57BL/6J mice, Sprague-Dawley rat, and AC16 cells	[72, 74]
	LIAS	C57BL/6J mice, Sprague-Dawley rat, and AC16 cells	[74, 77]
TCA Cycle Enzymes	DLAT	C57BL/6J mice	[10, 70]
	PDH/KGDH	No	[63, 78]
Fe-S Cluster-related	ISCU	No	[59, 66]
Oxidative Stress Pathways	NLRP3 Inflammasome	No	[8, 9]
	cGAS-STING	No	[64]
UPS System	UPS	C57BL/6J mice, Sprague-Dawley rat, H9c2 cells, and endothelial cells	[67, 68]

Abbreviations: ATP7A/ATP7B, P-type ATPases 7A/7B; CCS, copper chaperone for superoxide dismutase 1; cGAS-STING, cyclic guanosine monophosphate-adenosine monophosphate synthase-stimulator of interferon genes; LIAS, lipoic acid synthetase; PDH/KGDH, pyruvate dehydrogenase complex/alpha-ketoglutarate dehydrogenase complex; CTR1, copper transporter 1; DMT1, divalent metal transporter 1; ATOX1, antioxidant 1; COX17, cytochrome C oxidase copper chaperone 17; FDX1, ferredoxin 1; DLAT, dihydrolipoamide S-acetyltransferase; ISCU, Fe-S cluster assembly scaffold protein U; NLRP3, nucleotide-binding oligomerization domain-like receptor protein 3; cGAS-STING, cyclic guanosine monophosphate-adenosine monophosphate synthase-stimulator of interferon genes; UPS, ubiquitin-proteasome system.

**Fig. 1.**
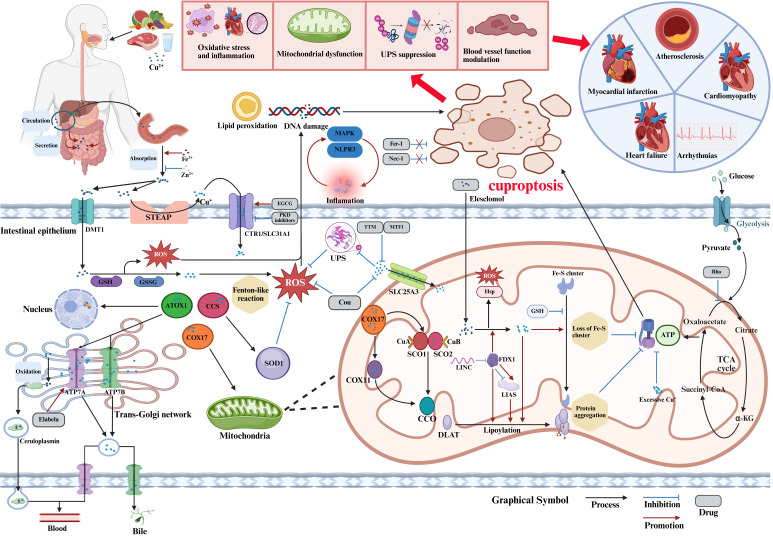
**Mechanisms of cuproptosis and its role in cardiovascular diseases**. Abbreviations: α-KG, α-ketoglutarate; ATOX1, antioxidant 1; ATP, adenosine triphosphate; CCS, superoxide dismutase copper chaperone; CCO, cytochrome C oxidase; CoA, coenzyme A; COX17/11, cytochrome C oxidase copper chaperone 17/11; Cou, coumarin; CTR1/SLC31A1, copper transporter 1/solute carrier family 31 member 1; DLAT, dihydrolipoamide S-acetyltransferase; DMT1, divalent metal transporter 1; DNA, deoxyribonucleic acid; EGCG, epigallocatechin gallate; FDX1, ferredoxin 1; Fe-S, iron-sulfur; Fer-1, ferrostatin-1; GSH, glutathione; GSSG, oxidized glutathione; HSP, heat shock protein; LIAS, lipoic acid synthetase; LINC, long intergenic non-protein coding RNA; MAPK, mitogen-activated protein kinase; MTF1, metal-regulatory transcription factor 1; Nec-1, necrostatin-1; NLRP3, nucleotide-binding oligomerization domain-like receptor protein 3; PKD, protein kinase D; ROS, reactive oxygen species; SCO1/2, cytochrome C oxidase assembly protein 1/2; SLC25A3, solute carrier family 25 member 3; SOD1, superoxide dismutase 1; STEAP, six-transmembrane epithelial antigen of the prostate; TCA, tricarboxylic acid; TTM, tetrathiomolybdate; UPS, ubiquitin-proteasome system.

Recently, in the development of models of FDX1 cytotoxicity, Schulz *et al*. [80] reported that FDX1 may function as a principal regulator of Cu^+^-induced cytotoxicity. Biochemically, they showed that as excess Cu^+^ damages FDX1 directly, the deficiency in Fe-S clusters and protein lipoylation resulting from this deficit in FDX1 contributes to failure of the ETC in mitochondria and the TCA cycle, which results in systemic metabolic collapse with a failure of ATP production and NADPH supply and insufficient α-ketoglutarate supply. Moreover, FDX1 deficiency also results in protein proteostasis stress through two parallel mechanisms: (1) the build-up of misfolded lipoylated proteins resulting from defective posttranslational modifications and (2) oxidative macromolecular damage induced by ROS overproduction [84]. Importantly, each of these disease mechanisms is synergistic in that mitochondrial failure increases ROS production, whereas proteotoxicity and oxidative stress reduce the ability to recover metabolism-leading to a positive feedback loop that eventually results in final metabolic shutdown and inflammatory activation, such as the putative last step of cuproptosis [64, 85]. This positive loop of metabolic breakdown might explain why dysfunctional tissue damage caused by Wilson’s disease is irreversible (Fig. 1), resulting in disordered copper accumulation.

## 3. Pathophysiological Role of Cuproptosis in CVDs

The regulatory network of cuproptosis plays a major role in the pathogenesis of CVDs via two interconnected processes: perturbed copper-induced redox imbalance and mitochondrial damage trigger cooperating inflammatory processes and proteostasis impairment characterized by the inhibition of the ubiquitin‒proteasome system (UPS), which exacerbates the imbalance in vasculature homeostasis. Understanding the results of these cascading events provides the possibility to intervene in CVD development by targeting therapy to their pathways.

### 3.1 Induction of Oxidative Stress and Inflammation

Preliminary evidence suggests that cuproptosis may contribute to CVD pathophysiology through oxidative stress pathways, although most supporting data derive from neuronal or cancer models, where redox disequilibrium and inflammation can contribute to disease propagation together [86]. At the heart of this process is FDX1-mediated Cu^+^ ions-dependent binding to lipoylated TCA cycle dehydrogenases, leading to disease-promoting protein aggregates and disruption of Fe-S cluster biogenesis. These dual actions lead to amplification of mitochondrial ETC failure and generation of ROS, forming a positive feedback loop to trigger the occurrence of cuproptotic cell death [87, 88]. The subsequent collapse of mitochondria releases mtDNA into the cytosol that triggers the cyclic guanosine monophosphate-adenosine monophosphate synthase-stimulator of interferon genes (*cGAS-STING*) signalling pathway to induce the inflammatory cascade—an important bridge connecting ROS with chronic inflammation [89]. Cumulatively, these cascades inflict macromolecular damage through lipid peroxidation, protein carbonylation, and DNA fragmentation, culminating in cardiomyocyte apoptosis, fibrotic myocardial remodelling, and atherosclerotic plaque development. These findings implicate cuproptosis as a potential contributor to CVD progression [87, 90].

Oxidative stress and inflammation are entwined reactions that amplify CVDs [91]. ROS originating from Cu^+^ ions accumulation can directly harm the endothelium and trigger inflammatory responses through the mitogen-activated protein kinase (MAPK) pathway and the formation of the inflammasome NLRP3 leading to cytokine IL/TNF release [92, 93]. Cytokines also amplify the oxidative stress since they trigger the increase in NADPH oxidase and the intracellular ROS overgeneration through the mitochondria. This feed-forward loop (between oxidant and inflammatory signals), amplifying and synergizing one with the other, sustains blood vessel injury/dysfunction and tissue impairment, propelling inexorable progression of the disease [94, 95].

### 3.2 Outcome of Mitochondrial Dysfunction

Mitochondria serve as the primary venue for cellular energy metabolism and the body’s cardiovascular homeostasis, and have critical requirements of physiological levels of Cu^2+^ as a cofactor for Complex IV’s involvement in the required functional metabolism of complex I [96, 97, 98]. Both insufficient and excessive Cu^2+^ ions levels disrupt mitochondrial homeostasis via distinct mechanisms, including impaired assembly of the essential Complex IV machinery [65, 99]; inadequate transport of Cu^2+^ ions to proteins required for complex IV structure causes structural problems and lower efficiency of oxidative phosphorylation. In contrast, cuproptosis is triggered by pathological copper overload that initiates the injury of cell membranes via lipid peroxidation, the direct inhibition of TCA cycle enzymes, and the depletion of Fe-S clusters, all in part due to the simultaneous collapse of protein homeostasis and Fe-S clusters synthesis [100, 101, 102]. The biological relevance of the mechanisms is illustrated for the cases of a Wilson’s disease model with limited levels of protein lipoylation and available Fe-S clusters content [10, 40]. Direct molecular evidence supports an implication where a Cu^+^ ions overbinding can induce the dysfunctional aggregation of lipoylated TCA enzymes [60], which fails to return to its catalytically active state. In addition, increased Cu^+^ ions dissolves coordination of metal cofactors in the electron transport chain, causing irreversible electron leakage, decrease of membrane potential and opening of permeability transition pores-all events that trigger bioenergetic arrest and PCD [21, 66, 103] (Fig. 1).

### 3.3 Modulation of Blood Vessel Function

Cuproptosis critically disrupts vascular homeostasis and drives CVDs pathogenesis through multilayered mechanisms [104, 105]. Excessive Cu^2+^ ion accumulation triggers vascular pathology by activating PCD and functional impairment in endothelial cells, leading to endothelial barrier dysfunction. This impairment facilitates the development of prothrombotic state, triggering an inflammatory environment, diminishing the vasodilation response-all these aspects play a significant role in the development of an atherosclerotic plaque [106]. A parallel pathological mechanism occurs in vascular smooth muscle cells (VSMCs), where cuproptosis induces phenotypic modulation characterized by reduced cellularity, impaired contractile-relaxation dynamics, and maladaptive remodelling [7]. These cellular-level defects are the mechanistic aetiology of hypertension and atherosclerotic lesion progression [107].

The subsequent pathophysiological cascade include the dysregulation due to Cu^2+^ ions of extracellular matrix (ECM), the accelerated matrix remodelling by accelerated degradation of collagen and elastin networks [108]. The final morphologic changes expressed as medial hypertrophy and luminal narrowing cause haemodynamic instability and loss of the vascular compliance. There is emerging evidence that a combination of Cu^2+^ ions induced oxidative stress, chronic inflammatory signalling and dysregulation of matrix metalloproteinase is involved in causing this remodelling process [109].

### 3.4 Suppression of the UPS

The UPS is a crucial control centre of intracellular proteostasis as it is responsible for the compartment-specific proteolysis for the stability of cellular function [110]. In addition to proteostasis, the UPS exerts crosstalk with endoplasmic reticulum stress responses to alter oxidative adaptation, PCD and differentiation-important biological processes of cardiovascular pathophysiology [111]. Recent studies demonstrate that copper overload disrupts UPS function via multiple mechanisms: specific copper inducers (such as CuET) and complexes including CuHQ inhibit proteasome activity or deubiquitinases, causing polyubiquitinated protein accumulation and impairing nuclear factor erythroid 2-related factor 1/Valosin-containing protein (*Nrf1*/p97) pathways [67, 112]. Cu^2+^ ions additionally facilitate GPX4 ubiquitination and oxidatively inactivates UPS proteins directly, thereby fuelling a positive loop of proteotoxic stress and apoptotic cell death [113, 114]. Cu^+^, whereas pathologically accumulated Cu^+^ reduces UPS function yet promotes cuproptosis, synergizing to precipitously accelerate proteostatic failure. Finally, continuous accumulation of Cu^+^ depletes UPS function, associated with defective protein clearance, in a context that is reported to be linked to cuproptosis and oxidative toxicity, triggering CVDs pathogenesis [68]. However, current evidence is limited to preclinical models, and human relevance requires further validation.

## 4. CVDs Related to Cuproptosis

Emerging evidence suggests that cuproptosis may contribute to the pathological development of CVDs with a mechanistic link of numerous underlying human pathologies. This review provides an overall pathophysiological view on how cuproptosis is involved in the mechanisms underlying the pathogenesis of atherosclerosis (AS), myocardial infarction (MI) and ischaemia-reperfusion injury (I/R), arrhythmogenesis, cardiomyopathy, maladaptive cardiac remodelling and heart failure (HF). In this way by breaking up these deeply intertwined pathological systems, we advocate the therapeutic potential of cuproptosis control and conceptualize integrative treatment schemes that synergize with management of the copper ion homeostasis and those of the more conventional cardiovascular treatment regimens.

### 4.1 Atherosclerosis (AS)

AS is characterized by the deposition of lipids, immune activation and endothelial dysfunction [115, 116], in which recent studies show that perturbed copper homeostasis is an important bridge connecting these pathology steps and elevated serum copper level has high concordance with the atherosclerotic load extent in the clinic [117]. Conversely, a panel of molecular analyses has suggested roles for transporter proteins such as SLC31A1 and SLC31A2, alongside antioxidant enzymes such as SOD1, as pivotal contributors to atherosclerotic progression, regulating the status of copper homeostasis, increased oxidative stress and pro-inflammatory activation of the vasculature [69]. Mechanistic studies indicate that cuproptosis may exacerbate endothelial dysfunction by impairing nitric oxide signalling and promoting leukocyte adhesion, suggesting a potential role in proatherogenic environment. Whether these pathways are clinically relevant in human CVDs requires direct evidence. This cell death pathway exacerbates endothelial dysfunction by impairing nitric oxide-mediated vasodilation and enhancing leukocyte adhesion, thereby promoting proatherogenic environment. The combined effect of copper dyshomeostasis, oxidation injury and inflammation signalling may represent a potential mechanistic pathway linked to atherogenesis, based on preclinical models and associative clinical data [106, 118].

Compartment-specific expression profiling of cuproptosis regulators in human atherosclerotic lesions spatial transcriptomics suggests expression of FDX1 and CRT1 are significantly enhanced in plaque-associated macrophages and VSMCs, while glutaminase (GLS) gene expression shows significant reduction, as part of a metabolic reprogramming associated with cuproptosis-mediated vasculature remodelling [70, 105]. Histochemical studies have moreover detected a stepwise increase of the redox-active metal ions in the chronic plaque, supporting their role in neointimal hyperplasia and matrix metalloproteases activity. *In vitro* studies prove the effectiveness of copper chelation in suppressing their effects [71]. A functional involvement of copper transport was emphasized by the colocalization of ATOX1 and ATP7A in arterial SMCs in atherosclerotic lesions. Inhibition of ATOX1 in the present study decreases ECM growth and supports enhanced plaque stability, placing this regulation axis directly in relation to disease pathology. These studies of mechanism are in harmony with epidemiology demonstrating the relationship between high dietary copper intakes and increased cardiovascular deaths [119]. However, further research is needed to account for potential confounding factors (e.g., socioeconomic status, concurrent nutrient intake) and establish causality.

### 4.2 MI and I/R

The mechanisms of MI and IRI are closely related to the deregulated cuproptosis-related genes (*CRGs*). Bioinformatics and animal studies show that ubiquitin-conjugating enzyme E2 D3 (Ube2d3) is upregulated in MI, exacerbating cardiomyocyte injury by increasing FDX1 and SLC31A1 expression and promoting neutrophil infiltration (Table 1) [72]. Upregulated *SLC31A1*, along with other *CRGs*, further aggravates myocardial damage by inducing mitochondrial dysfunction and recruiting monocytes [73]. Additionally, the protein levels of GLS and DLAT encoded by differentially expressed CRGs correlate with immune infiltration, metabolic alterations, and hypoxia pathways in MI [70]. However, such conclusions come only from bioinformatics analysis, with limited experimental validation.

Hypoxia and copper accumulation activate *FDX1* and *DLAT*, worsening oxidative stress and ventricular remodelling (Table 1) [74]. Clinical data confirm elevated serum copper in MI patients, associated with poor prognosis, while post-IRI copper release triggers aldehyde dehydrogenase 2 (ALDH2) degradation and FDX1-dependent lipoylation defects [120]. However, these molecular markers require validation in larger sample sizes.

### 4.3 Arrhythmias

Excess copper ions promote arrhythmias by disturbing cardiac electrophysiological activity, and redox imbalance and inflammation activation is also an associated pathophysiological basis in response to systemic copper excessive accumulation that is mechanistically related to cuproptosis [121]. Prior studies using *ex vivo* perfused rat heart models have demonstrated that excess copper catalyses free radical generation and exacerbates oxidative stress, thereby increasing the risk of arrhythmias such as ventricular fibrillation and impairing cardiac functional recovery. However, the copper chelator neocuproine can effectively mitigate this damage [122]. In addition, Hsiao *et al*. [123] confirmed that excessive copper exposure induces bradycardia and heartbeat irregularity in zebrafish embryos, whereas the glycyl-histidyl-lysine (GHK) tripeptide effectively mitigates cardiotoxicity through formation of a GHK-Cu chelate complex. Wilson’s disease is a genetic disorder caused by mutations in the *ATP7B* gene, leading to the dysregulation of copper metabolism due to impaired biliary copper excretion. Clinical evidence supports a correlation between Wilson’s disease and cardiac complications, such as ventricular fibrillation and tachycardia, which are likely due to copper-induced myocardial damage. These arrhythmias can even occur after the disease is cured by liver transplantation, as copper deposits remain in extrahepatic tissues [75, 76]. However, the current understanding of the association between cuproptosis and arrhythmias primarily revolves around systemic copper overload. Future investigations should elucidate whether cuproptosis directly disrupts cardiac ion channel function, thereby contributing to arrhythmogenesis, as this mechanistic link remains underexplored.

### 4.4 Cardiomyopathy

Recent bioinformatics analysis and partial cellular validation suggest that cuproptosis plays major roles in different types of cardiomyopathy, such as dilated, hypertrophic, arrhythmogenic right ventricular and diabetic cardiomyopathy. Molecular analyses invariably detect dysregulated target effectors of cuproptosis including *FDX1*, *SLC31A1*, and *DLAT*, with ATP7A mutations causing pathological copper accumulation with associated myocardial damage [124, 125, 126]. The combined role of hyperglycaemia and advanced glycation end-products also makes a permissive environment for the cuproptosis activation via FDX1-driven mitochondrial dysfunction and protein lipoylation defect [77]. The overlapping studies also provides insight into mechanistic cross-talk between cuproptosis and ferroptosis by either *GPX4*, or other antioxidant systems mediated via redox stress pathways during sepsis models [127, 128].

These mechanistic insights are exploited in current translational undertakings by employing therapeutic interventions: metallothionein provides protective effects against cardiac damage induced by doxorubicin through regulating intracellular copper bioavailability, and proprotein convertase subtilisin/kexin type 9 (PCSK9) antagonism conserves myocardial viability against I/R by stabilization of the *LIAS* pathway [129]. Specifically, diabetic cardiomyopathy appears to be particularly sensitive to therapeutic interventions that can influence copper homeostasis; new therapeutic approaches exploiting *SLC31A1*-mediated copper transport and DLAT aggregation promisingly appear to be able to improve fibrotic progression [130].

### 4.5 Cardiac Remodelling and HF

Cardiac remodelling refers to an adaptive or maladaptive molecular, cellular and interstitial rearrangement process in response to biomechanical stress or myocardial injury resulting in structural remodelling and functional deterioration [131]. HF is the end point of this process, and is classically described by symptomatology (dyspnoea and fatigue) and objective markers of cardiac dysfunction, such as natriuretic peptides and haemodynamic congestive markers [132]. During this remodelling, HF is established in a self-sustaining feedback loop. However, copper homeostasis acts in two complementary regulatory processes in cardiac physiology. Even though copper acts as a micronutrient important for maintaining a normal cardiac structure, deficiency as well as copper excess is paradoxically bidirectionally toxic to the heart. Notably, the observed cardiac effects of copper imbalance may exhibit species-specific variations, as most available data are derived from murine models. Murine models overloaded with copper present the eccentric ventricular remodelling feature with left ventricular ejection fraction enlargement and diminished thickness of the ventricular wall [133], although these phenotypic manifestations should be interpreted with caution when extrapolating to other mammalian species, including humans. The underlying mechanisms for copper supplementation-mediated improvement of pressure-overload-induced cardiac hypertrophy are not fully elucidated but may involve upregulation of myocardial vascular endothelial growth factor (VEGF) expression and promotion of coronary angiogenesis [134]. The content of cellular copper is tightly regulated by the coordination of several transport proteins, soluble chaperones and copper-dependent enzymes as well as transcriptional regulators [118]. Therefore, the tightly regulated homeostasis of copper is essential to preserve myocardial structure and electromechanical coupling.

Therapeutic copper supplementation reverses maladaptive hypertrophy induced by copper deficiency and alleviates associated pathological sequelae such as mitochondrial ultrastructural defects, sarcomeric disarray, and cytoplasmic vacuolization. Copper replenishment in the underloaded rodent model corrects the physiology of the failing heart through several mechanisms including rescue of defective excitation-contraction coupling by normalization of calcium handling, inhibition of inflammatory pathways, as well as altering ECM remodelling by transcriptional control [9, 78]. Importantly, copper excess can also display the same pathological phenotype seen in copper deficiency such as a failure of mitochondrial and contractile architecture integrity and metabolic dysfunction [135], which common end points lead to cardiomyocyte death despite different aetiological pathways. These bimodal toxicity seem to be leading to different ultimate end points by common upstream signalling pathways.

## 5. Therapeutic Strategies for Cuproptosis in CVDs

Elucidating the role of cuproptosis in CVDs provides a foundation for developing therapeutic strategies targeting this PCD. This section examines current advances in cuproptosis detection methodologies, pharmacological modulators (inducers and inhibitors), and clinical translation efforts, highlighting the potential of these approaches to guide future research and therapeutic interventions aimed at mitigating cuproptosis-driven cardiovascular injury.

### 5.1 Detection Methods

The detection approaches of cuproptosis in CVDs are based on the integration of combinatorial methods aimed at analyses of cellular phenotypes and molecular pathways analyses, in a complementary way that jointly uncovers the nature of cuproptosis mechanism [136]. *In vitro* experiments tend to focus on examining cell viability loss, growth arrest and apoptosis signals to validate the onset of cell death [10], while quantitatively analysing intracellular levels of copper ions for classifying the degree of cuproptosis [137]. Light microscopy and electron microscopy give visual evidence through phenotypic changes that are pathogenomic such as membrane rupture, mitochondrial swelling, endoplasmic reticulum enlargement and nuclear margination [7].

Biochemical profiling was carried out to elucidate the dysregulation of pathways involved in the process of cuproptosis execution [45], which is characterized by among others lipoylated DLAT aggregation, Fe-S proteins depletion and upregulated heat shock protein 70 (HSP70), which were identified using standard methods like quantitative polymerase chain reaction (PCR), western blots and immunohistochemistry (Table 1) [10, 136]. Meanwhile, metabolic assays have been applied to tracking enzyme activities and flux in pathways in oxidative stress, lipoic acid metabolism, TCA cycling and mitochondrial respiration for kinetic analyses to cuproptosis progression [138, 139, 140].

### 5.2 Inducers and Inhibitors of Cuproptosis

The new concept of cuproptosis as a PCD process provides opportunities for therapeutic intervention by selectively modulating cuproptosis inducers and modulators. The pharmacologic approaches to cuproptosis inhibition to date include five major types: copper chelators, mitochondrial respiration inhibitors, antioxidant network, transcriptional regulators and lncRNAs. Conversely, strategies to induce cuproptosis exploit mechanisms such as metal ionophores, modulators of membrane transporters, and inhibitors of TCA-cycle and mitochondrial bioenergetics [9, 114]. Copper chelation therapy has shown promise in preclinical models of CVDs, particularly in conditions involving cuproptosis. These agents exhibit varying binding affinities towards Cu^2+^ and Cu^+^ ions, depending on their structural arrangements and redox states. In clinical practice, tetrathiomolybdate (TTM) and trientine are used to reduce systemic copper overload. TTM exhibits low oral bioavailability (21%), a longer half-life (27 hours), and significantly increases serum copper concentrations [141]. TTM markedly inhibits intestinal copper absorption (by 82%) through the formation of stable copper-albumin complexes, reduces copper deposition in the liver and brain, and is particularly suitable for patients with neurological symptoms without significant cardiovascular side effects. However, it does not promote copper excretion, and long-term use may disrupt copper metabolic balance [23]. In contrast, trientine shows wide distribution after oral absorption, is primarily excreted renally, has a shorter half-life (2–4 hours), and requires multiple daily doses [142]. Trientine primarily reduces systemic copper burden by enhancing urinary copper excretion, making it suitable for chronic copper accumulation. However, it may cause cardiovascular reactions such as hypotension and carries a risk of worsening neurological symptoms [143]. Overall, TTM offers greater neuroprotective advantages, whereas trientine is more commonly used for long-term copper clearance but requires vigilance against its potential neurological and cardiovascular adverse effects. Additionally, these chelators decrease vascular copper availability, mitigating atherogenic pro-inflammatory effects in CVDs. TTM and trientine also suppress redox signalling by inhibiting ROS production [8], but its clinical relevance in human atherosclerosis remains to be established.

Concentric therapeutic approaches are aimed towards systemically tuning copper homeostasis. For instance, zinc acetate supplementation upregulates metallothionein expression in enterocyte gut tissues thereby minimizing systemic copper intake and deposition to liver [144]. Notably, metallothionein upregulation plays a vital role in treating diabetic cardiomyopathy by protecting the myocardium from oxidative stress, which is linked to disrupted copper homeostasis [145]. Mitochondrion-targeted strategies involve rhodamine-mediated suppression of ETCs and UK5099-induced inhibition of pyruvate transport, which reduces cellular sensitivity to cuproptosis [146, 147]. Antioxidant compounds coumarin derivatives and deferiprone have the abilities to act in tandem as chelators for the Cu^2+^ ions and ROS scavengers [148, 149, 150]. Flavonoids such as 5-hydroxyflavone and troxerutin exhibit potent protective effects on the cardiovascular system by blocking copper-driven Fenton reactions, inhibiting vascular smooth muscle proliferation, and suppressing NLRP3 inflammasome activation in AS models [151]. The transcription regulation by activation of a metal-responsive factor 1 provides stabilization of the intracellular metal homeostasis by induction of the expression of metallothionein [152]. Recent studies suggest that networks of noncoding RNAs regulate cuproptosis, showing an inverse correlation with genes in related pathways. Among these candidates, cancer susceptibility candidate 8 [153]. Recent studies show that Sirtuin 3 (SIRT3) controls the cuproptosis by regulating copper transporters and stabilizing FDX1, thereby reducing DLAT aggregation and mitochondrial damage in HF. SIRT3 ameliorates the DLAT aggregation and mitochondrial damages in HF but exacerbates cuproptosis in liver/kidney injuries [154].

To pharmacologically stimulate cuproptosis, different methods elevate intracellular concentration of Cu^2+^ ions or hinder the balance of copper homeostasis. Copper ionophores like elesclomol and disulfiram facilitate the cellular internalization of Cu^2+^ ion bypassed endogenous regulation to trigger cuproptosis [155]. Furthermore, natural substances like epigallocatechin gallate (EGCG) upregulate the expression of *SLC31A1*, which may enhance cytoplasmic accumulation of Cu^+^ ions. This mechanism is associated with improved cisplatin uptake in cancer cells, as demonstrated in non-small cell lung cancer models through ROS-dependent activation of the extracellular signal-regulated kinase 1/2 (ERK1/2) and nuclear paraspeckle assembly transcript 1 (NEAT1) pathway [156]. Ultra-small Cu nanoparticles (CuO-NPs) in CVDs models show a dose-dependent (low dose increases vascular reactivity, and high dose impairs cardiac inflammation) phenomenon with cautionary need of accurate clinical dosimetry [109, 157]. On the other hand, protein kinase D inhibitors like CID2011756, pentoxifylline downregulate the expression of the transporter ATP7A, leading to aggravation of intracellular Cu^+^ ions storage [156, 158], and the transcriptional activators like E26 transformation-specific transcription factor 3 (*ELF3*) and specificity protein 1 (*SP1*) upregulates the expression of *SLC31A1* to aggravate cuproptosis [159]. Noncoding RNAs networks also contribute to this regulatory framework. For example, LINC02362 modulates copper toxicity sensitivity by sponging miR-18a-5p to increase *FDX1* expression levels [160] (Fig. 1).

### 5.3 Clinical Application

The cuproptosis therapeutic concept presents a potential twofold clinical application within cancer and cardiovascular medicine. Anticancer therapeutic exploitation has shown compelling activity against pancreatic, breast and renal clear cell carcinoma by triggering a cooperative network of several potentiating mechanisms [161, 162, 163]. This method is applied as therapeutic approach by selectively addressing ATP7A activity in tumour tissue in order to locally accumulate Cu^+^ ions, creating cell-damaging environment due to redox imbalance, mitochondrial instability, and elicitation of pro-inflammatory signalling [164, 165]. In particular, such therapeutic approach synergistically improves classical immune-therapy treatment results by combining it with photothermal treatment regimes, bringing combinatorial therapeutic benefits.

Cardiovascular therapeutic strategies leverage copper chelation to address disease pathology. Preclinical studies suggest TTM may reduce Cu^2+^ levels and attenuate inflammatory pathways implicated in atherosclerotic progression, although its impact on oxidative stress remains unclear. However, clinical trial data validating these effects in patients with CVDs are currently lacking, highlighting the need for further translational research [166, 167]. Additional treatment modalities that mitigate pathogenic transport across the cell membrane opening new therapeutic windows for CVDs [9, 168]. Flavonoid-rich botanicals including hawthorn and scutellaria baicalensis ameliorate myocardial ischaemia via dual mechanisms: chelating excess copper and enhancing SOD1/MT2A antioxidant defences, validated in diabetic HF models [169, 170]. In contrast, controlled and selective copper ionophores exert specific proteotoxic stress in damaged cardiomyocytes and shows therapeutic potential in HF by inducing antioxidant defence as well as gene expression reprogramming [171].

Low-dose copper nanoparticles synergize with fish oil to enhance nitric oxide (NO) bioavailability and vasodilation in I/R although toxicity thresholds require rigorous monitoring [109]. Targeting cuproptosis represents a potential therapeutic avenue for CVDs; however, challenges such as tissue specificity and off-target effects must be addressed in future research. Peptide elabela decreased vascular calcification and mitochondrial dysfunction by activating *PPAR-γ* signalling and regulating copper homeostasis via ATP7A, thereby ameliorating cellular senescence and cuproptosis in VSMCs [172]. Merestinib stabilizes Fe-S clusters, inhibiting lipoylated protein aggregation and avoiding ROS damage as well as apoptosis. While Merestinib was originally investigated in hepatic injury models, the potential of Merestinib to correct metal ion balance via *NFE2L2* activation offers therapeutic potential in AS and I/R correlated to copper overload [173]. Traditional Chinese Medicine formulations including Gandou decoction rich in curcuminoids enhance biliary copper excretion and reduce cardiac fibrosis in Wilson’s disease-related HF, bridging herbal pharmacology with cuproptosis modulation [174]. Metallothionein as a scavenging metal protein helps doxorubicin-caused heart disease protection through modulating the cuproptosis pathway to stabilize the mitochondrion and balancing the redox [128]. Furthermore, SIRT7 sirtuin family member regulates copper-dependent cell death through the yes-associated protein (YAP)/ATP7A signalling to maintain mitochondrial homeostasis, becoming a potential target for hypertension-associated CVDs [175].

Moving forwards, rational targeting of PCD pathways with enhanced specificity by robust targeting systems and complementary/combinatorial therapeutic strategies becomes the focus for modern therapeutics development. However, significant knowledge gaps must be bridged to translate these approaches into clinical practice, including the need for direct evidence of cuproptosis in human cardiovascular tissues, a deeper understanding of its interplay with other cell death mechanisms, the development of tissue-specific copper modulators to avoid systemic toxicity, and large-scale clinical validation correlating serum copper levels and cuproptosis markers with patient outcomes. Combinations of pH-responsive nano delivery systems and molecularly targeted nanoparticles along with the mechanisms complementary therapeutic treatments are key emerging strategies to tackle the challenge of robust inhibition of PCD pathways with rational mechanisms. Taken together, these strategies boost therapeutic specificity and limit systemic toxicity with an optimized biodistribution profile and a drug combination pharmacological profile that was identified in more recent preclinical work [176, 177, 178]; however, clinical translation remains to be explored.

Notably, current investigations of cuproptosis in the treatment of CVDs remain considerably limited and are primarily confined to bioinformatics analysis and a small number of cell models. The overwhelming oncology orientation of this research field has resulted in a critical lack of clinical trials validating the efficacy and safety of cuproptosis-targeting interventions in patients with CVDs. Despite promising preclinical data, no copper-targeted therapies have been approved for CVDs, highlighting the need for human trials to evaluate safety and efficacy beyond copper metabolism disorders and some tumours. Although the underlying molecular mechanisms and therapeutic approaches have been demonstrated in cancer models, their cardiovascular-specific applicability, efficacy and safety require thorough evaluation.

## 6. Conclusions and Future Perspectives

Cuproptosis has emerged as a transformative concept in cardiovascular pathobiology, representing a PCD modality driven by dysregulation of mitochondrial copper homeostasis. This process is initiated through the copper-mediated aggregation of lipoylated enzymes and the destabilization of Fe-S clusters, which induce metabolic collapse by two interconnected mechanisms: the direct inhibition of TCA cycle enzymes and the secondary amplification of ROS due to compromised ETC integrity. Preliminary evidence suggests that the ensuing bioenergetic failure and redox imbalance initiate a self-perpetuating cycle of oxidative damage, mitochondrial membrane permeabilization, and endothelial‒mesenchymal transition, potentially accelerating the progression of CVDs. Preliminary studies have observed dysregulated expression of FDX1 and LIAS in some patients with CVDs, suggesting their potential as biomarkers, although further validation is required. However, it must be noted that although emerging studies implicate cuproptosis in CVD pathogenesis, the current evidence presents inconsistencies. Some clinical studies have reported elevated serum copper levels correlating with CVD severity, whereas others have found no significant association [8], possibly due to variations in study design, population characteristics, or copper measurement methodologies. Additionally, mechanistic insights primarily derive from preclinical models, and reproducibility across different experimental systems remains to be fully validated. These discrepancies highlight the need for standardized protocols and larger cohort studies to clarify the role of cuproptosis in clinical CVD endpoints.

As the recent work highlighted that cuproplasia is a copper-regulated proliferative process that could serve as a complimentary pathophysiologic function to cuproptosis. Different from the cell death caused by the over-excessive copper content in cuproptosis, cuproplasia is based on subtoxic copper levels through activating mitogenic signalling in forms of copper-responsive kinase cascades including MAPK/ERK and phosphatidylinositol 3-kinase/protein kinase B (PI3K/AKT), transcriptional networks (*NF-kB* and *HIF-1α*).While first observed in the cancer biology field, this proliferative cascade has in function an overlapping process with cuproptosis in the vascular diseases like AS where phenotypic switching of VSMCs is a dual regulatory axis of copper-mediated cellular dynamics (copper causes a balance between proliferative adaptation and terminal cell death during vascular remodelling) [13, 179, 180].

Physiological or pharmacological control of copper metabolism presents two options for CVDs protection. Clinically approved copper chelators, such as TTM and trientine, can exert therapeutic effects in specific settings. By sequestering labile copper, they may prevent cuproptosis while simultaneously inhibiting copper-dependent physiological processes, including proliferation, redox protection, and other copper-related functions. This nonelective effect underscores the paramount importance of the future generation of more specific modulators specifically of the protein component of the chaperone such as ATOX1 and CCS, and transporters including ATP7A and ATP7B. Remaining study areas relate to the spatiotemporal visualization of cardiovascular copper fluxes, copper epigenetic mechanisms controlling CRGs under haemodynamic perturbation and interactions in the cell death field. Experimental testing of the mechanistic paradigm of microenvironment modulations (changes in pH, hypoxic effects, glycocalyx health status) by differential cell sensitivity remains highly needed. Programmable metal microenvironments in emerging model systems like clustered regularly interspaced short palindromic repeats (CRISPR)-engineered copper sensing zebrafish and patient-derived organoids offer the possibility for powerful large-impact platform technologies to confront species-specific caveats and develop mechanistic knowledge of Copper-mediated disease pathophysiology.

Critical gaps remain in translating cuproptosis mechanisms to CVDs. Although key molecules such as FDX1 and LIAS are mechanistically compelling, they still lack validation in human cardiovascular tissues. Future studies should profile copper-dependent death signatures in biopsies from patients with CVDs, develop tissue-specific copper modulators to avoid systemic toxicity, and prioritize cohort studies correlating serum copper levels with cuproptosis markers and clinical outcomes. For next generation copper-targeted therapeutics in CVDs medicine, we suggest a multidimensional research strategy based on molecular cartography, dynamic tracking and selective conditioning. Specifically, molecular cartography is the mapping of CVDs metallomic networks with a molecular resolution synchrotron-based X-ray fluorescence microscopic with single-cell transcriptomic profiling. Dynamic monitoring requires developing activatable biosensors that track real-time copper speciation dynamics during disease progression. Precision modulation involves designing tissue-selective chelation systems using nanoparticles engineered to respond to microenvironmental redox states and pH variations.

In summary, cuproptosis shapes a new paradigm of cardiovascular copper biology that involves convergent mitochondrial bioenergetics, redox homeostasis and metalloprotein signalling pathways. The concept of cuproplasia elucidates the contextuality inherent in the dichotomy of copper effects in vasculature adaptation, but the preliminary evidence for cuproptosis-directed therapeutics suggests more translational potential by directly linking the drugs with cell death. In conclusion, growing evidence suggests an association between copper dysregulation and CVD progression, highlighting the need for comprehensive therapeutic strategies targeting this mechanism. Although preclinical data suggest that modulating copper homeostasis may influence CVD progression, translational studies are needed to determine whether copper levels can be actively targeted in clinical practice.
